# Association of county perinatal resources and gestational weight gain in West Virginia, United States

**DOI:** 10.1186/s12884-019-2650-7

**Published:** 2019-12-16

**Authors:** Wilson A. Koech, Christa L. Lilly

**Affiliations:** 1 Department of Epidemiology, School of Public Health, WV University Health Sciences Center, Morgantown, WV USA; 2 Department of Biostatistics, School of Public Health, WV University Health Sciences Center, Morgantown, WV USA

**Keywords:** Pregnancy, Gestational weight gain, Rural health, Perinatal resources

## Abstract

**Background:**

Inappropriate (inadequate or excessive) gestational weight gain (GWG) is of great concern to maternal, fetal and infant health. Different maternal and fetal risk factors are associated with GWG, but little is known about a more distal risk factor: inadequate county-level perinatal resources. Therefore, the study aim was to investigate GWG in women living in counties with below average perinatal resources in comparison with their counterparts living in counties with above average perinatal resources.

**Methods:**

Retrospective study of 406,792,010–2011 West Virginia births in 55 counties. The outcome was GWG and the main predictor was county perinatal resources. Hierarchical linear mixed model was used to investigate the association of county perinatal resources and GWG.

**Results:**

County perinatal resources was associated with GWG (*p* = 0.009), controlling for important covariates. Below average county perinatal resources was not significantly associated with a decrease in mean GWG (M: − 5.29 lbs., 95% CI: − 13.94, 3.35, *p* = 0.2086), in comparison with counties with above average county perinatal resources. There was significant difference between average, and above average county perinatal resources (M: − 17.20 lbs., 95% CI: − 22.94, − 11.47, *p* < 0.0001), controlling for smoking during pregnancy and other covariates.

**Conclusions:**

Average county perinatal resources was associated with reduced mean GWG relative to above average county perinatal resources, but not below average county perinatal resources. However, this could be due to the small number of counties with above average resources as the effect was in the hypothesized direction. This highlights one of the challenges in county perinatal resource studies.

## Background

Obesity among childbearing women is twice the numbers observed in mid 1970s in the United States, [1] and this can contribute to gestational weight gain (GWG) that is inadequate or excessive as per the American Institute of Medicine recommendation [2]. According to a recent study of 28 states in United States, approximately 21 and 47% of women had inadequate and excessive GWG respectively [3]. Inappropriate (inadequate or excessive) GWG is of great concern to maternal, fetal or infant health [4–6]. Past studies have associated inappropriate weight gain with maternal and child health issues; these health issues include gestational diabetes and hypertension, small for gestational age and large for gestational age neonates, birth complications, childhood obesity, postpartum pregnancy weight retention, neonatal morbidity and mortality [7–12]. Other risk factors poor prenatal care, poor nutrition, obesity, smoking during pregnancy, lower education levels, poor pregnancy weight management, and low income among other socio-ecological and psychosocial risk factors [13–16]. Even though numerous studies have investigated risk factors associated with GWG in United States (U.S), [3, 8, 12, 17] none of these studies is known to have been conducted in a state with predominantly rural or Appalachian population [18].

Additionally, no known study investigates county perinatal resources as a risk factor for GWG. County perinatal resources is a composite variable derived from resources that influences maternal child health at different perinatal stages following perinatal periods of risk approach [19]. One of these stages is maternal health and prematurity. Maternal health and prematurity can be influenced by risk factors such as chronic diseases, health behaviors, prenatal care, among others, and availability of county perinatal resources such as primary care providers and access to healthy food options might limit the impact of these risk factors on maternal well-being.

Therefore, the main objective of this study is to investigate the association between county perinatal resources and GWG in West Virginia (WV), a predominately-rural state in central Appalachia. Identifying risk factors associated with GWG will not only contribute new health information for better policy making and resource allocation, but the findings will also be vital for pregnancy weight management or pregnancy weight intervention, and pregnancy nutritional food intake. Studies in United States and other parts of the world suggests that GWG interventions is associated with reduction in excess GWG as well as a reduction of risk factors (for example gestational diabetes and hypertension) that are associated with adverse pregnancy outcomes [20–23].

## Materials and methods

### Population

Three data sources were used in this retrospective study. Retrospective study was favored instead of prospective study because data was readily available, it is less expensive, and study can be completed within a shorter period. Data sources were: (1) De-identified linked birth/death vital health data records for WV 2010–2011 [24], (2) United States Census Bureau website, [25] and (3) County health ranking and roadmaps website [26].

The West Virginia University Institutional Review Board (IRB) approved this study.

### Measures

The overall goal of this study was to investigate an association of county perinatal resources and GWG in WV, a predominately rural state in central Appalachia. West Virginia Bureau for Public Health, Charleston, WV provided de-identified 2010–2011 linked birth/death data. The number of births for a two-year period was 41,176 births in 55 counties. After removing univariate outliers for GWG, as well as exclusion of observations with missing maternal age, missing gestational age, and missing birth weight, 41,106 observations remained.

Percentage of county population in poverty in 2010–2011 was extracted from U.S. Census Bureau [25]. Percentage of county zip codes with health food as well as number of primary care providers in 2010–2011 were extracted from County health rankings and roadmaps website [26]. All these three datasets were linked together by county of residence.

Study variables included individual-level and global-level characteristics. Individual-level characteristics were birth identification number, birth year, gestational hypertension, infant’s sex, insurance type/payer, maternal education level, smoking during pregnancy, gestational diabetes, trimester prenatal care began, GWG, maternal age, and race/ethnicity. Global level variables included county of residence, county’s perinatal resources, county’s medium household income, percentage of county population in poverty, county population estimate, number of county primary care providers, and percentage of county zip codes with access to healthy food options.

### Outcome

The outcome of interest was a continuous variable called GWG (in lbs.) reported in linked birth vital health data records. GWG is maternal weight gain during pregnancy, which is the difference between pregnancy weight (i.e. maternal weight before birth) and pre-pregnancy weight. Healthcare providers collects maternal pregnancy weight during prenatal visits.

### Predictor

The primary predictor of interest was county perinatal resources which is a composite categorical variable (0 = Above Average, 1 = Average, 2 = Below Average). Number of primary care providers in a county and percentage of county zip codes with healthy food options were used to derive categorical county perinatal resource levels. Primary health care providers were identified as appropriate if they met or exceeded Solucient recommendation of approximately 23 primary care providers per 100,000 population, and not appropriate otherwise [27]. Zip codes have been used in the past to study disparities in access to healthy food options [28, 29]. Healthy food options was defined as having at least 50% of county zip code with access to healthy food options (e.g., supermarkets). Access to healthy food options was defined as grocery stores with greater than 4 employees with fresh fruits and vegetables stands as provided by county health ranking and roadmaps [26]. When census tracts are typically used to identify food deserts, at least 33% must have limited access to healthy food options [30]. Although food desert can be defined as counties with at least 50% of its population having limited access to health food options, [31] 50% is used in this study to describe access of health food options to minimize overestimation of the findings.

Thus, county perinatal resources were obtained by combining percentage of county zip codes with access to healthy food options and number of primary care providers in a county as follows: Counties with equal to or less than 50% of zip codes with access to healthy food options, and with less than recommended number of primary care providers was coded as having below average perinatal resources. Counties with equal to or less than 50% of zip codes with access to healthy food options and with more than recommended number of primary care providers, or vice versa was coded as having average perinatal resources. Finally, counties with more than 50% of zip codes with access to healthy food options, and with more than recommended number of primary care providers was coded as having above average perinatal resources.

### Covariates

Covariates included categorical and continuous variables. Categorical covariates included gestational hypertension, neonate’s sex, smoking during pregnancy, race/ethnicity, trimester prenatal care began, and insurance type. Continuous covariates included, percentage of county population in poverty, and maternal age (years).

### Statistical methods

Data management and analysis used in this paper was SAS/STAT software, version 9.4 of SAS systems for windows [32]. Descriptive statistics are presented to explain measures of central tendency and spread for continuous variables as well as proportion and frequency distribution for categorical variables. Additionally, inferential statistics was conducted in order to produce population parameters for hypotheses testing using hierarchical linear models (HLM) using mixed modeling procedure with random effects as described by Suzuki and Sheu, and Bell and his colleagues [33, 34]. HLM is recommended for nested data.

Regression assumptions were tested during statistical modeling process. Before deriving a parsimonious model, continuous variables were tested for bivariate associations using Pearson’s correlation. Furthermore, categorical variables were assessed for bivariate associations using Spearman’s correlation. Most covariates were not correlated except weak correlation between insurance type and maternal education level, and between smoking during pregnancy and maternal education level (both had *r* = − 0.3). Strong correlation existed between county median household income and percentage of county population in poverty (*r* = − 0.8). Thus, education and median household income were dropped from the model due to concerns about multicollinearity. All other regression model assumptions held.

A final parsimonious model was obtained by first, removal of all interactions with *p*-value greater than 0.1, and second, removing all interactions at once with *p*-value greater than 0.05. The second step above was repeated until no interaction had *p*-value greater than 0.05. Finally, main effects removed if they had *p*-value greater than 0.05. The best fitting model was selected via Akaike information criterion (AIC) goodness-of-fit, taking into account variability due to nesting as assessed by calculating intraclass coefficient (ICC). Fully parameterized HLM was a better fit than intercept only HLM (AIC = 251,985, ICC = 0.05 and AIC = 203,919, ICC = 0.07 respectively. HLM was used to capture differences between different levels of county perinatal resources. Final model results, after assumption testing, are presented. These results include omnibus test, parameter estimates, standard errors, confidence intervals, t-statistics, and *p*-values.

## Results

### Descriptive statistics

Births meeting inclusion criteria in WV in 2010–2011 period were 41,106. Infants living in counties classified as above average perinatal resources included 288 infants in 1 county in 2010, and 6710 infants in 8 counties in 2011. Infants living in counties classified as average perinatal resources included 17,938 infants in 41 counties in 2010, and 12,185 infants in 32 counties in 2011. Additionally, infants living in counties classified as below average perinatal resources included 2204 infants in 13 counties in 2010, and 1781 infants in 15 counties in 2011 (Table 1).

**Table 1:** Births characteristics in West Virginia United States per county perinatal resource, *n* = 41,106

County Perinatal Resource Category	2010		2011		GWG (lbs.)	
	County Count	Births	County Count	Births	Mean	SD
Below average	13	2204	15	1781	27.4	13.8
Average	41	17,938	32	12,185	27.6	14.1
Above average	1	288	8	6710	27	14.6

*GWG* Gestational weight gain, *SD* standard deviation

Consistent with rural Appalachian demographics, majority of the population were White Non-Hispanic. When separated by county-level resources, 92.5, 93.8 and 98.4% of births were White Non-Hispanic residing in counties with above average perinatal resources, counties with average perinatal resources, and counties with below average perinatal resources respectively. Among women who reported their insurance information, approximately 62.8% were documented using Medicaid health insurance in counties with below average perinatal resources, while about 60% had at least high school graduate. See Table 2 for more details.

**Table 2:** Comparison of gestational weight gain and county perinatal resource by maternal and infant characteristi\cs in West Virginia United States, 2010–2011 births. *n* = 40,679

Variable	Category	All participants	GWG	County perinatal resource			*p*-value
			Mean (SD)	Above Average	Average	Below Average	
		N (%)	Pounds	N (%)	N (%)	N (%)	
Highest Education Level	≤ High School	21,460 (53.5%)	29.9(14.4)	2936 (50.4%)	13,427 (55.0%)	2139 (60.1%)	<.000^a^
	BS or some college	14,966 (37.3%)	28.2(13.9)	2430 (41.7%)	8555 (35.0%)	1260 (35.4%)	
	≥ Master’s degree	3678 (9.2%)	28.7(13.3)	458 (7.9%)	2452 (10.0%)	161 (4.5%)	
Gestational Hypertension	No	37,184 (92.1%)	29.3(14.1)	5194 (89.2%)	22,438 (91.8%)	3241 (91.0%)	.000^b^
	Yes	3181 (7.9%)	29.4(14.6)	630 (10.8%)	1996 (8.2%)	319 (9.0%)	
Sex	Male	20,776 (51.1%)	27.8(14.4)	3051 (52.4%)	12,316 (50.4%)	1869 (52.5%)	.995^b^
	Female	19,903 (48.9%)	27.2(13.9)	2773 (47.6%)	12,118 (49.6%)	1691 (47.5%)	
Insurance Type	Non-Medicaid	14,383 (41.0%)	27.9(13.5)	2524 (43.3%)	10,177 (41.7%)	1326 (37.2%)	<.000^b^
	Medicaid	20,699 (59%)	26.6(14.3)	3300 (56.7%)	14,257 (58.3%)	2234 (62.8%)	
Smoke during pregnancy	No	29,936 (73.8%)	28.1(13.9)	4422 (75.9%)	17,947 (73.5%)	2509 (70.5%)	<.000^b^
	Yes	10,633 (26.2%)	25.8(14.6)	1402 (24.1%)	6487 (26.5%)	1051 (29.5%)	
Gestational Diabetes	No	38,763 (96.0%)	27.7(14.1)	5561 (95.5%)	23,328 (95.5%)	3395 (95.4%)	.493^b^
	Yes	1602 (4.0%)	22.7(14.1)	263 (4.5%)	1106 (4.5%)	165 (4.6%)	
Trimester prenatal care started	1st Trimester	32,640 (83.5%)	27.9(14.0)	4976 (85.4%)	20,315 (83.1%)	2905 (81.6%)	<.000^a^
	2rd Trimester	5247 (13.4%)	25.8(13.8)	703 (12.1%)	3419 (14.0%)	498 (14.0%)	
	3rd Trimester	1006 (2.6%)	25.0(14.7)	123 (2.1%)	612 (2.5%)	140 (3.9%)	
	No Care	206 (0.5%)	16.3(16.3)	22 (0.4%)	88 (0.4%)	17 (0.5%)	
Race/ethnicity	Other	2403 (5.9%)	26.1(14.2)	437 (7.5%)	1519 (6.2%)	58 (1.6%)	<.000^b^
	White Non-Hispanic	38,248 (94.1%)	27.6(14.1)	5387 (92.5%)	22,915 (93.8%)	3502 (98.4%)	
Gestational Weight gain (lbs.)	Mean(SD)	27.5 (14.2)	–	27.0 (14.6)	27.6 (14.1)	27.4 (13.8)	.013^**c**^
Maternal Age (years)	Mean(SD)	26.2 (5.9)	–	26.6 (5.9)	26.2 (5.9)	25.7 (5.7)	<.000^**c**^
County Population in poverty (%)	Mean(SD)	18.5 (4.1)	–	17.4 (2.9)	18.4 (4.2)	21.1 (3.7)	<.000^**c**^

*SD* Standard deviation of the mean, *GWG* Gestational weight gain

*N(%)* = Frequency and percentage relative to the row variable

^a^ = Cochran-Mantel-Haenszel chi-square statistics’ *p*-value for null hypothesis test of no association

^b^ = Two-sided *p*-value for Cochran-Armitage trend test, null hypothesis test of no trend in column proportion

^**c**^ = F-statistics’ *p*-value for null hypothesis test of no difference in means

### HLM model

The study’s primary research aim was to investigate GWG difference in women living in counties with below average perinatal resources in comparison with their counterparts living in counties with above average perinatal resources.

Hierarchical linear model was applied and county perinatal resource means compared. Variables that were significantly associated with GWG included individual level effects, county level effects and interactions effects. Significant individual level effects maternal age F (1, 24,000) = 150.0, *p* < .0001, gestational hypertension F (1, 53) = 40.2, *p* < .0001), sex F (1, 54) = 24.1, *p* < .0001), insurance status F (1, 54) = 6.5, *p* = .0109),smoking during pregnancy F (1, 54) = 66.3, *p* < .0001), gestational diabetes F (1, 54) = 106.5, *p* < .0001), trimester prenatal care began F (1, 141) = 38.3, *p* < .0001, and race/ethnicity F (1, 50) = 8.8, *p* < .0001. County level effects were county perinatal resource F (2, 13) = 30.4, *p* < .0001, and percentage of county population in poverty F (1, 24,000) = 14.4, *p* = .0001, and interactions effects were interaction of smoking during pregnancy and insurance status F (1, 50) = 5.6, *p* = .0223, interactions of percentage of county population in poverty and county perinatal resource F (2, 24,000) = 41.9, *p* < .0001, and interaction of maternal age and insurance status F (1, 24,000) = 13.9, *p* = .0002.

Table 3 demonstrate relationship between referent category and average GWG. On average, women living in counties with below average county perinatal resources had approximately 5.3 pounds less GWG compared to women living in counties with above average county perinatal resources, holding other variables in the model constant (t = − 1.3, *p* = .2086). On the other hand, women living in counties with average county perinatal resources had an average of 17.2 pounds less GWG, compared to women living in counties with above average county perinatal resources, holding other variables in the model constant (t = − 6.5, *p* < .0001). Additionally, for every unit increase on percentage of county population in poverty and below average county perinatal resources interaction, GWG increases by an average of 0.8 pounds, holding other variables in the model constant (t = 3.7, *p* = .0002).

**Table 3:** Significant individual level and county level characteristics associated with gestational weight gain in West Virginia United States, 2010–2011 births. *n* = 24,502

Effect	Comparison Category	e	σ	95% Confidence Limits		t^a^	*p*-value
				Lower	Upper		
Intercept		42.51	0.05	35.94	49.09	12.96	<.0001
County perinatal resources (ref = above average)	Average	−17.20	0.05	−22.94	−11.47	−6.48	<.0001
	Below average	−5.29	0.05	−13.94	3.35	−1.32	0.2086
Mother’s Age (years)		−0.14	0.05	−0.19	−0.09	−5.48	<.0001
% of county population in poverty		−1.01	0.05	−1.34	−0.67	−5.94	<.0001
Gestational Hypertension? (ref = No)	Yes	1.94	0.05	1.33	2.55	6.34	<.0001
Newborn’s Sex (ref = Female)	Male	0.85	0.05	0.50	1.20	4.91	<.0001
Insurance/Payer (ref = Non Medicaid)	Medicaid	1.75	0.05	−0.09	3.59	1.91	0.0614
Smoked during pregnancy? (ref = No)	Yes	−2.70	0.05	−3.62	−1.78	−5.90	<.0001
Gestational Diabetes? (Ref = No)	Yes	−4.32	0.05	−5.17	− 3.48	− 10.32	<.0001
Trimester prenatal care began (ref = 1st Trimester)	2nd Trimester	−1.62	0.05	−2.12	−1.11	−6.32	<.0001
	3rd Trimester	−2.53	0.05	−3.58	−1.49	−4.81	<.0001
	No care	−10.36	0.05	−12.97	−7.74	−7.82	<.0001
Race/Ethnicity	White non-Hispanic	1.12	0.05	0.36	1.88	2.97	0.0046
Smoked during pregnancy? and Insurance/payer interaction	Yes vs Medicaid	1.21	0.05	0.18	2.24	2.36	0.0221
% of county population in poverty and County perinatal resources interaction	% in poverty*Average	1.24	0.05	0.95	1.53	8.40	<.0001
	% in poverty* Below average	0.76	0.05	0.36	1.17	3.73	0.0002
Mother’s Age and Insurance/Payer interaction	Mother’s Age * Medicaid	−0.12	0.05	−0.19	−0.06	−3.72	0.0002

*e* estimate, **σ** = standard error of the estimate

^a^ = t-statistics

Smoking during pregnancy is associated with average reduction of GWG by 2.7 pounds in comparison with non-smokers during pregnancy, holding other variables in the model constant (t = − 5.0, *p*-value < .0001). Moreover, gestational hypertension is associated with increase in GWG by 1.9 pounds on average, in comparison with absence of gestational hypertension, holding other variables in the model constant (t = − 5.9, *p*-value < .0001).

## Discussion

The study was to determine association of county perinatal resources and maternal GWG. The hypothesis was that maternal GWG is significantly lower in counties with below average perinatal resources, in comparison with counties with above average perinatal resources. Identifying risk factors associated with GWG will not only contribute new health information for better policy making and resource allocation, but the findings will also be vital for pregnancy weight management or pregnancy weight intervention, and pregnancy nutritional food intake. Studies in the United States and other parts of the world suggests that GWG interventions is associated with reduction in excess GWG as well as a reduction of risk factors (for example gestational diabetes and hypertension) that are associated with adverse pregnancy outcomes [20–23].

The study findings show that GWG for women living in counties with below average resources was not statistically significant from women living in counties with above average resources. However, the effect appeared to show that GWG was lower for women in below average perinatal resource counties (− 5.3lbs), compared to higher perinatal resource counties, after controlling for other variables in the model. These findings, although not statistically significant for the below average counties, were consistent with various studies that associates poor perinatal resources (for example nutrition and prenatal care) with poor perinatal outcomes [35, 36]. In their study at Sheba Medical Center Israel, Abu-Saad and Fraser established that low social economic status is a vehicle for unhealthy exposures that might affect normal fetal development, [37] but in the United States, most women of poor socioeconomic status often qualify for health supplemental nutritional programs. These health supplemental programs include women, infants and children (WIC), Medicaid, and supplemental nutritional assistance through supplemental nutritional assistance program (SNAP). Such health and nutritional programs might be a much-needed intervention for prenatal care and access to enough food during pregnancy. In this study, approximately 63% births in counties with below average perinatal resources were identified to be utilizing Medicaid, therefore majority of the women are more likely to be beneficiaries of WIC and SNAP, hence a greater likelihood of GWG improvement. This is emphasized by the study findings, which indicated an average increase in GWG by 0.8 pounds when percentage of county population in poverty and county perinatal resources interact. Therefore, this finding is corroborated by findings in previous studies, [37–39] that highlights the importance of having good access to healthy food and access to primary care physicians. In different global literature review, resource distribution is identified as a significant factor associated with poor pregnancy outcomes [39, 40]. In addition, Wu and colleagues noted that women exposed to poor nutrition are likely to have poor fetal growth and development [41].

Some of the significant covariates, such as percent of county in poverty, is interesting to further examine. The study findings show that as proportion of population living in poverty increases in counties with limited perinatal resources (below average), women tend to gain almost a pound of GWG on average (*p* = 0.0002), controlling for other variables in the model. This result seems odd because one might expect less GWG as more people live in poverty, but women might be experiencing some protective effect. For instance, Medicaid has been suggested as having a protective effect among populations living in poverty [42].

Other important covariates were smoking during pregnancy and trimester prenatal care began. The relative importance of both smoking during pregnancy and trimester prenatal care began with GWG was not surprising, since several studies have shown that smoking cessation during pregnancy and earlier prenatal care are important for healthy fetal growth [43, 44]. Women smoking during pregnancy had an average of 2.5 pounds less, compared to women not smoking during pregnancy, while women not attending prenatal care had an average of 10.4 pounds less compared to those who stated their prenatal care in the first trimester.

Despite the various statistically significant associations in these findings, there are a few possible limitations. (1) The data used were collected for other purposes other than research; therefore, the findings might be inherent with information bias, (2) Difficulty in accounting for seamless flow of population between counties. Even though statistical method was utilized to account for county data interdependence, ease in movement of county residence can facilitate utilization of adjacent county resources, especially by population in poor counties. Nevertheless, this phenomenon only biases the association towards the null; therefore, the findings are more conservative. (3) All births were included in the study. Births other than singleton might bias GWG, but GWG histogram followed a bell shape without any noticeable skewedness. (4) Limitation on weighting scale reliability in different hospitals, and how GWG weight is measured is potential source of error, even though self-reported weight during pregnancy is not common. In addition, GWG can vary greatly based on the trimester of the initial prenatal visit and the birth date. Such variability can be another source of error, and so as the birth date variability.

Regardless of these limitations, the study outcomes contribute essential health information that can be used to guide healthcare professional and policy makers in GWG management and resource allocation. Future studies should explore barriers that hinder county resource allocation for the reduction of maternal fetal unhealthy exposures.

## Conclusion

Study findings adds unique perspective on the importance of state resource allocation. Even though, below average county perinatal resources were not significantly associated GWG in comparison with above average county resources, greater attention should be paid to counties with below average perinatal resources because the interaction between county perinatal resources and county poverty distribution was identified. Percentage of county population in poverty and below average county perinatal resource interaction was found to be significantly associated with GWG. In addition, average county perinatal resources were significantly associated with GWG in comparison with above average county resources. Important covariates that warrant the attention of policy makers include smoking during pregnancy and trimester prenatal care began.

## Data Availability

The linked birth/death data that support the findings of this study are available from the West Virginia Bureau for Public Health, Charleston WV, but restrictions apply to the availability of these data, which were used under license for the current study, and so are not publicly available. Data are however available from the authors upon reasonable request and with permission of the West Virginia Bureau for Public Health, Charleston WV. The following link contains contact information for the West Virginia Bureau for Public Health. http://www.wvdhhr.org/bph/hsc/ContactUs.asp. County Health Ranking and Roadmaps data are available to the public online and interested parties can access this data using the following link; https://www.countyhealthrankings.org/app/west-virginia/2019/downloads. The last data source is available to the public through the United states Census website - https://www.census.gov/programs-surveys/saipe/data/datasets.2010.html.

## Abbreviations

AIC: Akaike information criterion
GWG: Gestational weight gain
HLM: Hierarchical linear models
ICC: Intraclass coefficient
SNAP: Supplemental nutritional assistance program
WIC: Women, infants and children
